# Caring from behind the face mask in healthcare: Learning from the dramatic arts

**DOI:** 10.1007/s40037-021-00691-8

**Published:** 2021-11-04

**Authors:** Paul Murphy, Dearbhail Lewis, Gerard J. Gormley

**Affiliations:** 1 School of Arts, English and Languages, Queen’s University Belfast, Belfast, UK; 2 Belfast Health and Social Care Trust, Belfast, UK; 3 Centre for Medical Education, Queen’s University Belfast, Belfast, UK

**Keywords:** COVID-19, Pandemic, Simulation, Care, Masks, Gestures

## Abstract

The COVID-19 pandemic has made its impact across the globe with great voracity. New routines have displaced older more established ones with ruthless efficiency—no more so than in healthcare. In meeting these challenges, many healthcare workers have had to prepare for and enact many new ways of working. Regardless of their speciality or stage of training, health professions educators (HPEs) have helped train our healthcare workforce in developing new skills with great tempo. Throughout all of these efforts one constant has guided our endeavours—the humane connection with those that *provide* and those that *seek *healthcare.

However, with COVID-19 we have had to distance ourselves from our patients, and colleagues, and clad ourselves in various items of personal protection equipment (PPE). The protective barrier also acts as a barrier to personal interaction and therefore presents challenges in how we connect with each other on a humane level. Few disciplines have engaged with the complexities of verbal and gestural communication as thoroughly and consistently as the dramatic arts. Actors in Ancient Greece would perform wearing masks and used oratory as well as gestural communication to enrapture the audience.

Drawing upon the dramatic arts, we aim to explore the relationship between face and mask and thereby provide reflective insights for HPEs to help guide healthcare workers in their communication from behind the face mask.

## Introduction

The COVID-19 pandemic has made its impact across the globe with great relentlessness. We have seen unprecedented changes to how we work and how we live, and no more so than in the field of healthcare. In the words of Dame Clare Marx, Chair of the General Medical Council (GMC) of the United Kingdom: *“None of us has experience of a pandemic like this. Dealing with coronavirus is the biggest challenge to face the NHS since it was founded. And it’s going to ask a lot of us all.”* [1].

**Fig. 1:**
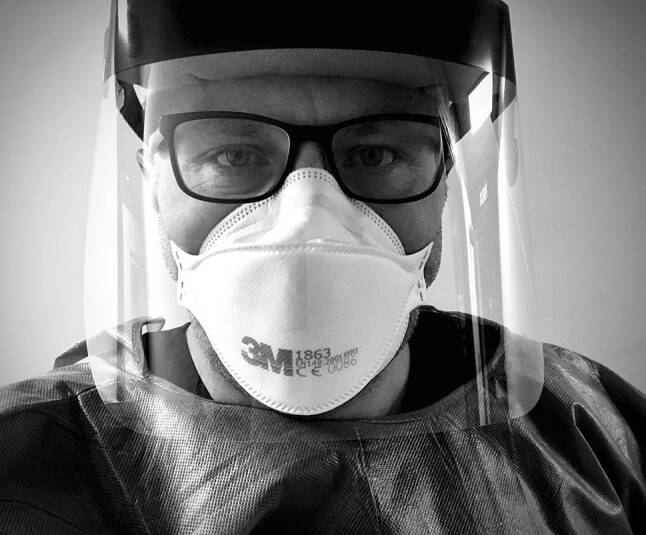
Example of a healthcare worker wearing PPE and the view often presented to a patient. Copyright held by the authors

In meeting this challenge, many healthcare workers (HCWs) have had to prepare for and enact many new ways of working. Regardless of their speciality or stage of training, health professions educators (HPEs) have helped train our HCWs in developing new skills with great tempo. Throughout these efforts one constant has guided our endeavours—the humane connection with those that provide and those that seek healthcare. However, with COVID-19 we have had to distance ourselves from our patients, and colleagues, and clad ourselves in various items of personal protection equipment (PPE). The protective barrier also acts as a barrier to personal interaction and therefore presents challenges in how we connect with each other on a humane level (Fig. 1).

Verbal communication allows us not only to convey clinical information but also express empathy. Aside from the content of our verbal communication, there are many dimensions to effective communication, so the emphasis is not just on *what* we say but *how* we say it [2, 3]. The sincerity of what we say is important in our conversations with patients and important to emphasise in health professions education. Facial gestures contribute significantly to our communications, be that the slight smile when expressing that a blood pressure is normal or the concerned expression when breaking bad news to a patient. In the context of caring for patients with COVID-19, PPE can create barriers for communication and shared sense-making. From seeing the masked face of a doctor when being intubated in ICU, to discussing treatment options with a distressed patient, such facial gestures are often taken for granted. Nonetheless, those gestures we normally consider mundane are incredibly complex. As HPEs we must instil the importance of the humane connection between patients and our learners. Furthermore, we must help learners develop new ways to effectively communicate behind barriers like face masks.

Few disciplines have engaged with the complexities of verbal and gestural communication as thoroughly and consistently as the dramatic arts. Actors in Ancient Greece would perform wearing masks and used oratory as well as gestural communication to enrapture the audience [4]. Across the centuries costume would enhance the defining qualities of characters in plays from Sophocles to Shakespeare [5].

In the modern era advances in actor training have enabled increasingly realistic and compelling performances where the nuances of verbal and gestural communication can be conveyed in exquisite detail [6]. We may be forced to wear face masks, but we must learn to care from behind the face mask and be seen, by the patient, to be doing so. Drawing upon the dramatic arts, we aim to explore the relationship between face and mask and thereby provide reflective insights for HPEs.

## Face-work, impression management and the dramaturgical metaphor

Each of us has a face that we deem unique and the key to our identity as sovereign individuals—although it is merely one amongst a myriad of faces. However, the face, like the self, is only defined and given meaning through social interaction—by seeing and being seen by others. Goffman’s ‘On Face-work’ built upon Durkheim’s study of religion in his analyses of interaction rituals in everyday life *‘*through whose symbolic component the actor shows how worthy he [sic] is of respect or how worthy he feels others are of it*’* [7, p. 19]. An individual’s face is a ‘sacred thing’, and the ‘expressive order’ required to sustain it is ‘a ritual one’ [7, p. 19]. By practising ‘face-work’ and helping others to avoid losing face, an individual protects the dignity and therefore the emotions of others. Goffman’s ‘Embarrassment and Social Organization’ continues the theme of face-work and describes the ‘gestures’ that provide the individual with ‘screens to hide behind’ when ‘discomfiture arises’ while ‘he [sic] tries to bring his feelings back into tempo and himself back into play’ [8, p. 266]. The notion of face-work is of course based on the premise that the interactants can see (or feel) each other’s face and interpret their respective gestures and manage their impressions accordingly. Goffman further evolves his notion of face-work in *The Presentation of Self in Everyday Life* and begins the book with the contention that when an individual is in the ‘presence of others’ they will ‘have to act’ so that they *‘*intentionally or unintentionally express’ themselves, and ‘the others will in turn have to be impressed by’ them [9, p. 2]. Goffman outlines a theory of impression management based on the dramaturgical metaphor where individuals present versions of themselves to make an impression on their fellow interactants like an actor performing a role in a play. The emotional dimension of social interaction that Goffman examines is an essential and often overlooked aspect of communicating with patients in the dual sense of the emotional impression made by the HCW on the patient and the emotional impression made upon the HCW during challenging conversations.

## Adapting to the face mask through configurations of the whole body

In the COVID-19 pandemic the face mask worn by HCWs is first and foremost a protective barrier that delimits rather than accentuates impression management. HCWs wearing a face mask and visor have two barriers that constrain their voice as well as covering the lower half of their face. The physical facilities available to an HCW to compensate in this context include the eyes, eyebrows, and tilt of the head which can be used as part of gestural communication. The hands and arms can be used more than usual to accentuate messaging in conjunction with signs that are held or worn upon the person. In Ancient Greek theatre the mask ‘covered the whole head’ and the actor ‘brought the mask to life through configurations of the whole body’ [4, p. 110]. The voice can be projected by increasing its volume and fewer words can be chosen to simplify the message. Facial gestures can be exaggerated to emphasize key words and to accentuate indications of concern for the patient and empathy for their condition.

More importantly, the role of rehearsal in the dramatic arts can be brought more emphatically into HCP training. The dramatic arts are increasingly involved in healthcare education [10, 11], but the practice of engaging healthcare students and for that matter HCPs in the role of the patient in peer simulation is still relatively nascent [12]. In peer simulation, HCPs could act in the role of the patient that is normally accorded to simulated participants, in concert with other HCPs performing their respective professional role, as they try to communicate wearing various kinds of PPE. The intersection of theatrical rehearsal and healthcare simulation where role-play can offer a safer space in which HCPs perform as patients to avoid the obvious discomfort of, for example, expecting a patient in ICU to complete a questionnaire or engage in a focus group after they have been discharged to better understand their experience. Theatrical performance is not of course the same as simulated participation in healthcare education [13]. Nonetheless, the productive possibilities of engaging with the nuanced approach inherent to theatre rehearsal regarding voice and movement in relation to peer simulation merits further investigation [14]. Research could be conducted on the relative merits of different kinds of verbal and gestural techniques demonstrated by the HCPs that can be measured empirically.

## Caring through the face mask: reducing virus transmission but not compassion

In 2014, Seale et al. published the results of a qualitative study examining the attitudes of HCWs towards the use of face masks and respirators [15]. They found that participants felt that the use of face masks/respirators to be ‘another barrier’, and that communication with patients became more difficult. Respondents also identified that they felt uneasy about inducing anxiety in patients whilst using face masks or respirators. These experiences and feelings are common to many of us currently treating patients during the COVID-19 pandemic.

**Fig. 2:**
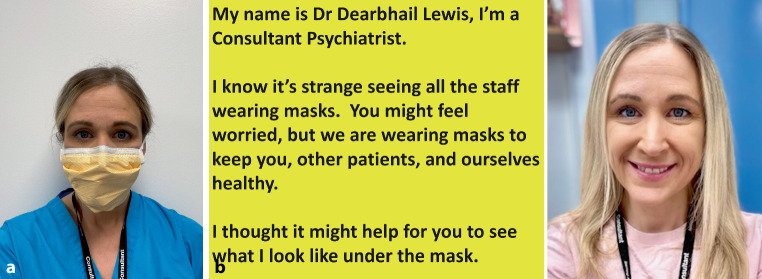
Example of an intervention to ‘reveal’ the person behind the face mask. Copyright held by the authors

More so now than ever, we remain keen to optimise communication and reduce anxiety experienced by our patients. One way of facilitating this is to allow patients to see what we look like under the face mask. To ensure that infection prevention and control measures are met, we must explore options other than allowing this by removal of the mask during patient contact. A card printed with the smiling face of the HCW can be presented to each individual patient. The card can also explicitly recognize the patient’s anxiety or distress, as demonstrated in Fig. 2.

The card can be used by the patient as a visual reminder of the humanity of the HCW in whom they are expected to place their trust. The patient can then either keep the card or it can be disposed of at the end of the interaction according to necessary infection prevention and control measures.

**Fig. 3:**
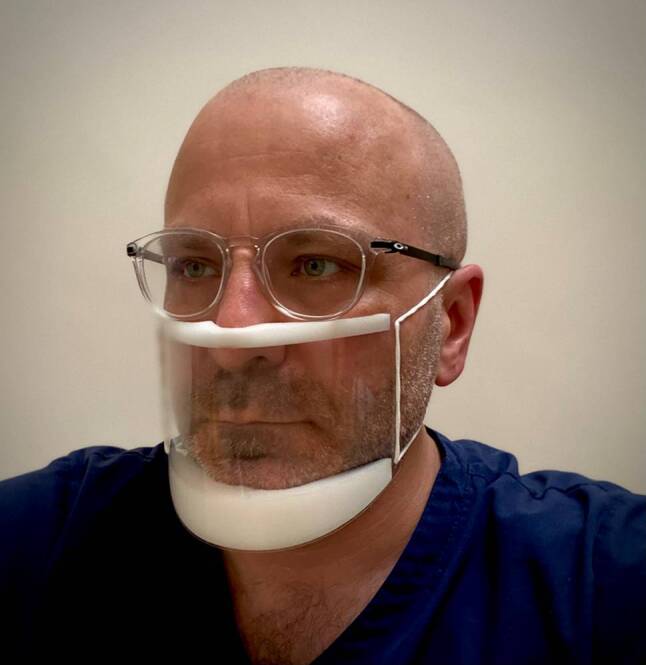
Example of a clear face mask than can permit a patient to see the facial gestures of their healthcare provider. Copyright held by the authors

Face masks have been developed to aid communication between patients who have a hearing impairment and their healthcare provider. As demonstrated in Fig. 3, clear face masks permit patients to see their healthcare providers face whilst reducing infection transmission. Whilst primarily designed to facilitate lip-reading for those patients with a hearing impairment, such transparent face masks can also allow other patients to see the healthcare provider’s facial gestures and expressions.

In addition to these techniques, there are many other ways in which healthcare providers can enhance their ability to connect with their patients; from printing their name on their visor or a having their photograph placed on a badge on their scrubs. There are of course many *body work* [16] practices which HCWs can finesse to express empathy. From the head tilt to the side, keeping eye contact and nodding appropriately variously demonstrate active listening to a patient’s concerns. Hand gestures can provide a powerful means of expressing empathy. Whether using hands to gesticulate as a HCW speaks to a patient, or gestures such as placing a hand over your heart as a sincere expression of empathy, such methods all reach out from behind the cladding of PPE to connect with our patients.

## Conclusion

We must not forget that medicine, however well based in science, is also a humane activity that must be founded on a continuity of care and compassion. Whilst many advances have been made on the technical front, HCWs have also strived to overcome many barriers to connect humanely with our patients. As HPEs it is important that we raise the awareness of the challenges of connecting with patients from behind PPE. Facial gestures are often considered mundane but are extraordinarily important to effective communication. Moreover, as HPEs we need to help guide our learners in developing new ways of connecting with their patients from behind PPE and honour their commitment to person-centred care by not only conveying empathy in *what* we say but how we *express* it. In addition to verbally communicating empathy, the use of other techniques such as ‘body work’—whether expressing with hands or head movements—may further help convey sincerity. Equally practical measures such as ‘friendly identification cards’ may also move towards improving the expression of empathy from behind PPE.

## References

[1] Marx C. Doctors will stop at nothing to provide care in this crisis—our job is to support them. 2020. https://www.gmc-uk.org/news/news-archive/doctors-will-stop-at-nothing-to-provide-care-in-this-crisis-our-job-is-to-support-them. Accessed 2 May 2020.

[2] Hall E (1959). The silent language.

[3] Birdwhistell R (1970). Kinesics and context: essays on body motion communication.

[4] Wiles D (2000). Greek theatre performance: an introduction.

[5] Barbieri D (2017). Costume in performance: materiality, culture, and the body.

[6] Benedetti J (2007). The art of the actor.

[7] Goffman E (1955). On face-work. Psychiatry.

[8] Goffman E (1956). Embarrassment and social organization. Am J Sociol.

[9] Goffman E (1956). The presentation of self in everyday life.

[10] De La Croix A, Rose C, Wildig E, Willson S (2011). Arts-based learning in medical education: the students’ perspective. Med Educ.

[11] Walsh I, Murphy P (2017). Healtheatre: drama and medicine in concert. Healthcare.

[12] Dalwood N, Bowles KA, Williams C, Morgan P, Pritchard S, Blackstock F (2020). Students as patients: a systematic review of peer simulation in health care professional education. Med Educ.

[13] Pritchard SA, Denning T, Keating JL, Blackstock FC, Nestel D (2020). “It’s not an acting job … Don’t underestimate what a&nbsp;simulated patient does”: a qualitative study exploring the perspectives of simulated patients in health professions education. Simul Healthc.

[14] Pritchard SA, Dalwood N, Keating JL, Nestel D, Te M, Blackstock F (2021). ‘It’s the ultimate observer role … you’re feeling and seeing what’s happening to you’: students’ experiences of peer simulation. BMJ Simul Technol Enhanc Learn.

[15] Seale H, Leem J-S, Gallard J (2015). ‘“The cookie monster muffler”: perceptions and behaviours of hospital healthcare workers around the use of masks and respirators in the hospital setting. Int J Infect Control.

[16] Twigg J, Wolkowitz C, Cohen RL, Nettleton S (2011). Body work in health and social care: critical themes, new agendas.

